# First record of *Zombrus bicolor* (Enderlein) (Hymenoptera, Braconidae, Doryctinae) in Western Europe

**DOI:** 10.3897/zookeys.219.3439

**Published:** 2012-09-04

**Authors:** Augusto Loni, Robert Spooner Hart, Andrea Lucchi

**Affiliations:** 1 Department of Tree Science, Entomology and Plant Pathology, Entomology Section, University of Pisa, Via San Michele degli Scalzi 2, 56124 Pisa, Italy; 2 University of Western Sydney, School for Science and Health, Locked Bag 1797, Penrith NSW, 2751 Australia

**Keywords:** Vineyard, new finding, wood borer parasitoid, exotic species, Anoplophora chinensis

## Abstract

The finding of *Zombrus bicolor* (Enderlein) (Hymenoptera: Braconidae: Doryctinae) in a Tuscan vineyard of the Siena province (Italy) represents the first record of this species in western Europe. A female was captured in summer 2009 with a malaise trap located in an organic vineyard. Until this finding, the species was recorded only in the Oriental regions of continental China, Taiwan, Korea and Japan and, very recently, in the eastern and southern parts of the Palaearctic region.

## Introduction

A single female of *Zombrus bicolor* (Enderlein) (Hymenoptera: Braconidae: Doryctinae) was collected in June 2009 in a malaise trap located in a vineyard (cv Sangiovese) of an organic farm in the Montalcino district (Siena-Tuscany-Italy, 43°05.21N, 11°28.51E). The trap was installed at ca 180 m above sea level in a typical rural Tuscan landscape, with vineyards included in rolling, gentle hills surrounded by cultivated grassland areas, woods and strips of shrubs, and wild bushes of deciduous trees. Until the current finding, *Zombrus bicolor* had only been reported as an Eastern Palaearctic and Oriental species, based on the description of specimens collected in Taiwan, China, Mongolia, Japan, Korea and, very recently, in Kyrgyzstan and in the European part of Russia (Astrakhan province) (Enderlein 1912; Fahringer 1929; Fischer 1980; Chou 1981; Papp 2003, Belokobylskij and Maetô 2009; Belokobylskij and Samartsev 2011).

The type specimens of *Zombrus bicolor* are two males stored in the collection of the Museum of the Zoology Institute in Warsaw (Poland). These males were collected by H. Sauter in 1907 in Takao (Formosa), the old name of the current city of Kaohsiung in south-western Taiwan, and subsequently described by Enderlein (1912).

**Figure 1.**
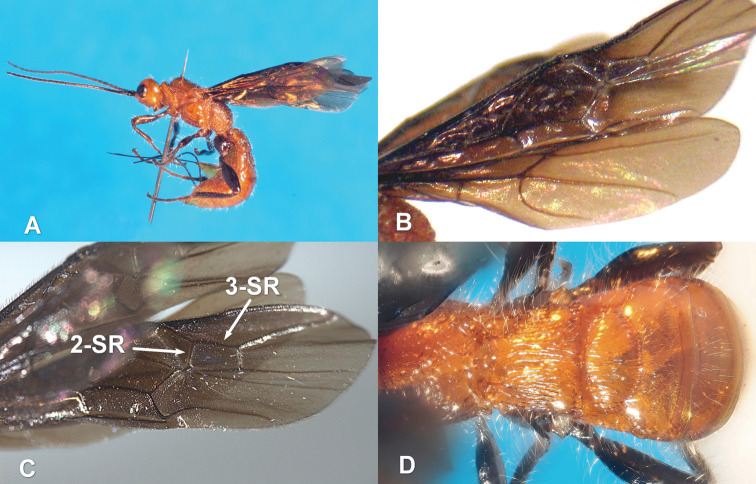
*Zombrus bicolor* female: **A** body lateral view **B** fore and hind wings **C** detail on the fore wing **D** abdominal terga 1-3 dorsal view.

*Zombrus bicolor* (Fig. 1A) belongs to the subtribe Odontobraconina of the tribe Holcobraconini, a monophyletic group including six genera and currently divided in the three subtribes Holcobraconina, Odontobraconina and Ivondroviina, (Belokobylskij 1992). The morphological features that characterize the tribe, the genus *Zombrus* and the species *Zombrus bicolor* are well described in Belokobylskij and Samartsev (2011).

**Figure 2.**
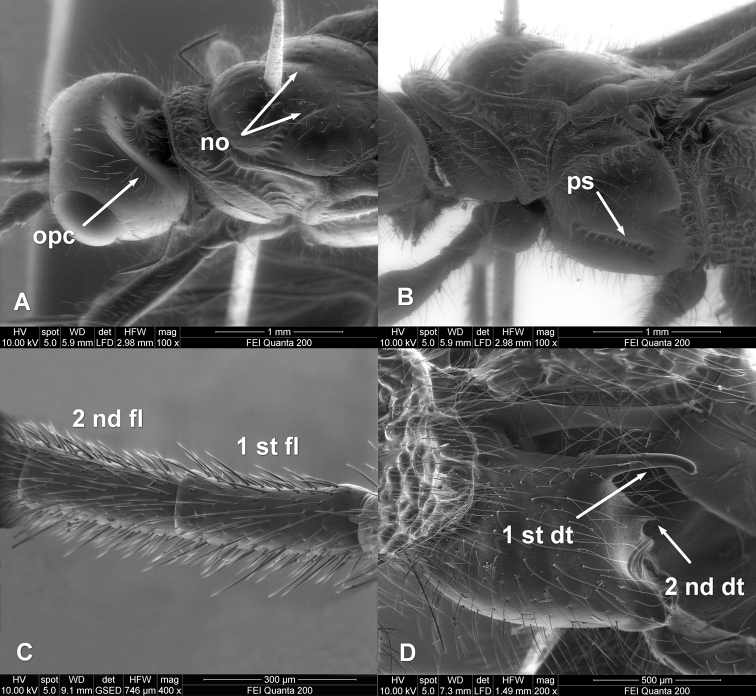
*Zombrus bicolor* female, SEM micrographs details on: **A** head and mesoscutum **B** mesopleuron **C** first and second flagellomeres **D** hind coxa. Abbreviations: **2-SR** and **3-SR** sectio radii veins of fore wing **opc** = occipital carina **no** = notauli **ps** = precoxal sulcus **1**^st^ and **2**^nd^ **fl** = first and second flagellomeres **1**^st^ and **2**^nd^ **dt** = first and second dorsal teeth of hind coxa. Morphological terminology is used according to van Achterberg (1993).

According to these authors, the typical morphological features of *Zombrus bicolor*, which distinguish this species from the other Palaearctic species of *Zombrus*, are the completely dark fore wings (Fig. 1B), the body covered with long and dense setae (Fig. 1 D, Fig. 2 A, B, D) and the presence of the occipital carina dorsally and, partly, laterally (Fig. 2A).

All the features of the specimen we collected in Italy match well with those reported by Belokobylskij and Samartsev (2011), with exception of the notauli which are not complete but reduced posteriorly on the mesoscutum (Fig. 2A).

Our finding could represent one of the many cases of accidental introduction of exotic insects that have occurred in Italy in the last decades. At European level as well, the accidental introduction of new insect species from their original areas has become more and more frequent and alarming. Intensification of plant trade with geographically distant commercial partners and global warming, that allows aliens to establish successfully in new ecosystems, are considered the main causes of the occurrence of invasive species.

As an important crossroad in the Mediterranean basin, Italy seems particularly suitable to the introduction and settlement of alien species, also because its landmass extends over a wide range of latitudes climatically suitable to harbour subtropical species in the South and insects coming from temperate and continental zones in the northern and central regions of the peninsula.

It has been estimated that about 200 exotic species have been introduced and established in Italy since 1970 (Longo 2009), the vast majority of whom are insect pests.

Importantly, our finding concerns not a pest but a parasitoid, which in this case could have followed his victim/s in the transfer from one continent to another. This is permissible every time that the natural enemy is a cryptic species, such as an endoparasitoid, or an ectoparasitoid living in concealed galleries excavated by larvae of wood boring insects.

*Zombrus bicolor* is known as a solitary, larval parasitoid of many wood boring beetles (Yu et al. 2010; Belokobylskij and Samartsev 2011), including *Anoplophora chinensis* (Forster) (Coleoptera: Cerambycidae) (Smith et al. 2007). This wood borer was first detected in 2000 in northern Italy, in the surroundings of Milan and rapidly became a serious threat to deciduous trees in urban areas and natural forests throughout northern and central Italy. *Anoplophora glabribennis* was also found in the same area in 2007 (Maspero et al. 2007).These species are major pests of many deciduous trees and fruit plants in their native Asian countries (Hu et al. 2009), are considered quarantine pests for the European Union according to the Directive 2000/29/EC and are subjected to monitoring and eradication programs (Herard et al. 2009).

Though data are not available at the moment on the presence of *Anoplophora chinensis* in the areas where we collected *Zombrus bicolor*, we cannot exclude that the braconid, in its movement to West Europe, had been carried by these or other specific hidden hosts.
